# An automated, data‐driven approach to children's social dynamics in space and time

**DOI:** 10.1111/cdep.12495

**Published:** 2023-12-08

**Authors:** Lisa Horn, Márton Karsai, Gabriela Markova

**Affiliations:** ^1^ Department of Behavioral and Cognitive Biology University of Vienna Vienna Austria; ^2^ Department of Network and Data Science Central European University Vienna Austria; ^3^ Alfréd Rényi Institute of Mathematics Budapest Hungary; ^4^ Department of Developmental and Educational Psychology University of Vienna Vienna Austria; ^5^ Institute for Early Life Care Paracelsus Medical University Salzburg Austria

**Keywords:** group dynamics, preschool peer groups, wearable sensor technology

## Abstract

Most children first enter social groups of peers in preschool. In this context, children use movement as a social tool, resulting in distinctive proximity patterns in space and synchrony with others over time. However, the social implications of children's movements with peers in space and time are difficult to determine due to the difficulty of acquiring reliable data during natural interactions. In this article, we review research demonstrating that proximity and synchrony are important indicators of affiliation among preschoolers and highlight challenges in this line of research. We then argue for the advantages of using wearable sensor technology and machine learning analytics to quantify social movement. This technological and analytical advancement provides an unprecedented view of complex social interactions among preschoolers in natural settings, and can help integrate young children's movements with others in space and time into a coherent interaction framework.

**Abbreviations**
ANN: Artificial neural networks
RFID: radio‐frequency identification
UWB: ultra‐wideband

From birth, children are embedded in a complex web of social relationships. Their earliest social interactions with caregivers are primarily dyadic, but their social network grows steadily to include multiple interaction partners when they enter complex social groups of peers in preschool (2–6 years). These peer groups are structured by affiliative relationships between individuals (e.g., friendships; Santos et al., 2015) and social dynamics within the group (e.g., segregation by common interests or gender; Martin et al., 2013), which are important for children's well‐being and classroom engagement (Chen et al., 2019; Rose et al., 2022). Yet for more than five decades, capturing the interactions in these complex social networks has been a challenge for researchers studying child development (Santos & Vaughn, 2018). Most live observations and video recordings fail to simultaneously capture the social interactions of multiple children (cf. Banarjee et al., 2023), and researchers typically face a trade‐off between recording the behavior of one child or a dyad in detail and restricting the data collection of multiple children to simplified measures. These challenges have severely limited the collection of large‐scale data sets of children's natural social behavior in complex social environments, leaving untapped potentially important sources of knowledge that could inform theories about early peer interactions and relationships.

As a solution to these challenges, we propose a framework based on automated, data‐driven analysis of children's movement (see Figure 1). Children often use movement as a social tool. They approach or withdraw from other individuals at specific velocities (Banarjee et al., 2023), resulting in distinctive proximity patterns in space (e.g., Santos et al., 2015). They also coordinate their movement with others—a concept referred to as “interpersonal synchrony” (Miles et al., 2009, p. 585)—in ways that may result in distinctive synchrony patterns over time (Fujiwara et al., 2020). The dimensions of space and time are likely closely interrelated with each other and across different levels, such as the individual, the dyad, and the group.

**FIGURE 1:**
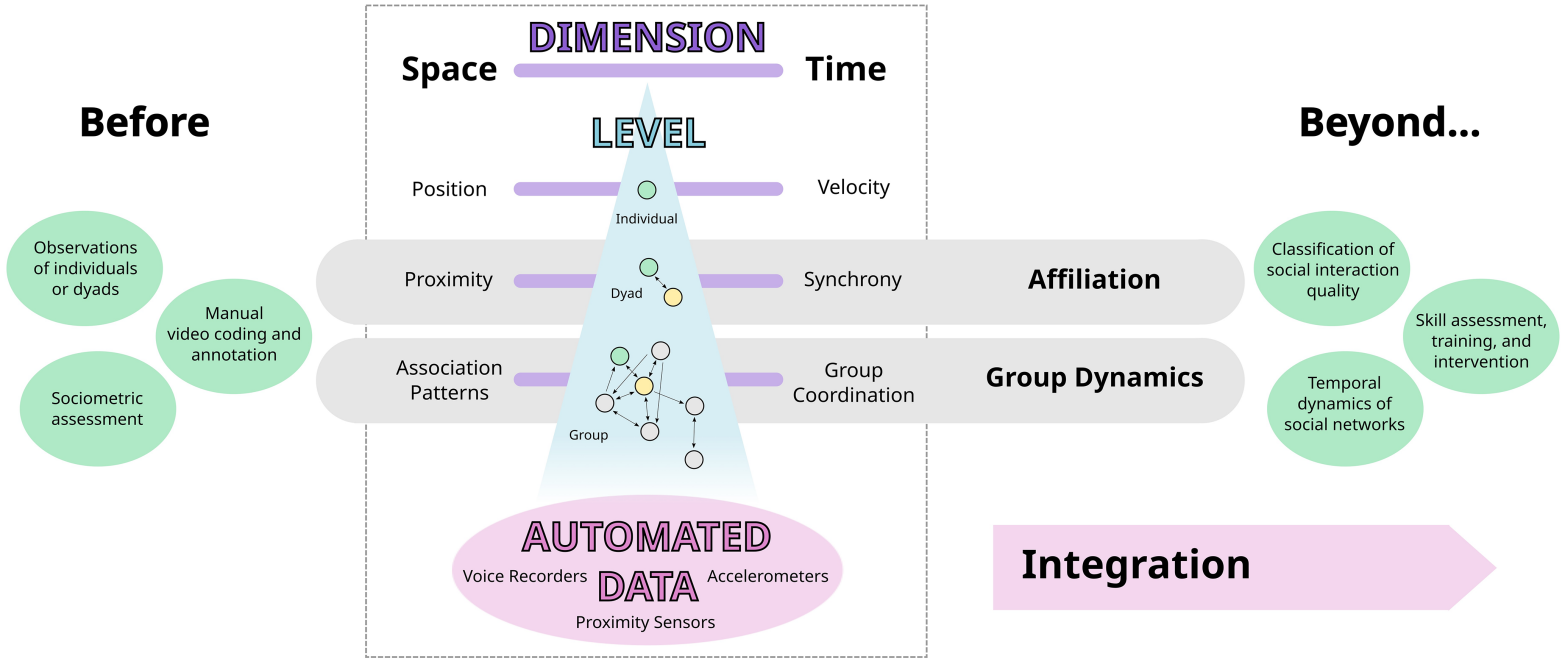
Architecture of the proposed conceptual framework. Most current research on affiliation and social group dynamics among preschoolers in natural settings relies primarily on human observation. Based on automated, data‐driven analysis of children's movement, we can gain insights that integrate the dimensions of space and time and the levels of the individual, the dyad, and the group. Machine learning can improve our classification of social interactions, opening opportunities for personalized assessment, training, and intervention.[Color figure can be viewed at wileyonlinelibrary.com]

In this article, we first review research demonstrating that proximity and synchrony contain rich information about social connections among 2‐ to 6‐year‐olds. We then highlight challenges in these lines of research, and argue for the advantages of using wearable sensor technology and machine learning analytics to quantify social movement. Finally, we integrate this information into a conceptual framework that can be used to reliably quantify complex social interactions among preschool peers in natural settings. Given the interdependence between novel data collection approaches and theory advancement (see Huber, 2009), the framework can also advance theoretical models of children's social development. For a comprehensive reporting of sociodemographic information about the participants in all research reviewed herein, see Table S1.

## PROXIMITY AND SYNCHRONY ARE IMPORTANT INDICATORS OF AFFILIATION AMONG PRESCHOOLERS

Independent locomotion is an important milestone in children's development (Campos et al., 2000). It ensures mobility and allows exploration of the environment (Goldfield, 1995). Beyond exploration, movement drives various developmental achievements, including language, cognition, and social communication (e.g., Campos et al., 2000; Piek et al., 2008). Moreover, children's movement with and in relation to others is indicative of their relationships (Banarjee et al., 2023; Messinger et al., 2019). Most prominently, proximity to and synchrony with others contain rich information about the quality of interactions and are thus very reliable indicators of affiliative relationships at preschool age and beyond (e.g., proximity: Hartup et al., 1988; Santos et al., 2015; synchrony: Rabinowitch & Knafo‐Noam, 2015; Tunçgenç & Cohen, 2016).

Spatial proximity has long been used as an objective measure to reliably detect affiliative relationships in groups of preschool peers (Howes, 1990). Young children spend more time in close proximity to peers they nominate as friends than in close proximity to peers they consider nonfriends (Hartup et al., 1988; Masters & Furman, 1981). Children use spatial proximity to infer friendship between two interaction partners, but only when the two individuals actively choose to be in proximity (Afshordi, 2019; Liberman & Shaw, 2019). Detailed analyses of proximity patterns on the group level reveal a stratification of preschoolers' social networks, with functionally distinct subgroups: Groups of friends are characterized by high levels of proximity among all members of the subgroup, whereas subgroups with lower levels of transitive proximity are more likely formed to achieve temporary goals (Santos et al., 2015). However, simplified measures of proximity, such as Euclidean distance, allow only a limited interpretation of complex social interactions. Because children actively choose to approach or move away from others (Tremblay et al., 1981), detailed observations of children initiating and terminating proximity, along with measures of interaction quality (e.g., approach speed), contain valuable information about preschool children’s social relationships (Banarjee et al., 2023), but we lack such systematic analyses of preschoolers’ dynamic adjustment of proximity to others in natural settings.

In addition to proximity, the temporal structure of children's movements as they unfold over time in relation to others' movements, so‐called interpersonal synchrony, also contains rich information about their social relationships (e.g., Feldman, 2012; Xu et al., 2020). In several studies, affiliation was reflected in higher levels of synchrony between interaction partners (Fujiwara et al., 2020; Latif et al., 2014). For example, 14‐month‐olds' behavior was more closely synchronized with their own mothers than with a female stranger (Bernieri et al., 1988). In other studies, toddlers spontaneously synchronized their locomotor activity with their mothers during free play (Hoch et al., 2021) and made inferences about others' affiliation based on movement synchrony (Cirelli et al., 2016; Fawcett & Tunçgenç, 2017). At the same time, experiencing synchrony has positive effects on affiliation among children, such as increased bonding (Tunçgenç & Cohen, 2016) and enhanced perception of similarity and closeness (Rabinowitch & Knafo‐Noam, 2015). These findings indicate that interpersonal synchrony may, in fact, play an important role in peer interactions. However, no studies have examined whether preschool children spontaneously synchronize their movements in natural settings.

## CHALLENGES IN ASSESSING PROXIMITY AND SYNCHRONY AMONG PRESCHOOL PEERS

As we have noted, preschool children's movements in space and time in relation to peers' movements can indicate their affiliation and social dynamics within natural groups. However, despite their demonstrated importance, the dimensions of space and time are rarely investigated together to provide a more complete picture of these complex social dynamics. For example, proximity patterns of preschoolers in natural groups are affected by children's movement over time. Simultaneously recording individual children's position and velocity would provide a more fine‐grained understanding of their exploration and use of space. Analyzing multiple children's coordinated movement to a new location and how they re‐establish proximity would offer valuable insight into their relationships and social dynamics. Furthermore, most developmental psychological research uses the individual as the unit of analysis. With recent methodological advances, psychologists have begun to consider the level of the dyad and even the group in their analyses (e.g., Coco & Dale, 2014). However, these different levels are usually examined in isolation, leaving in the dark the dynamic interplay among individual, dyad, and group behavior (see Figure 1).

The main reason for these challenges may be the difficulty of acquiring reliable and valid data in both space and time from more than two peers during natural interactions. Traditionally, research quantifying the movement of multiple individuals along the dimensions of space and time has relied primarily on live or recorded observations. These methods are not only costly in terms of resources and time, but they are also prone to errors and subject to interpretation bias, particularly when tracking complex movements and interactions. The challenges we have described can be addressed by using lightweight, wearable sensor technology and machine learning analytics to quantify preschoolers' social movement with their peers.

## AUTOMATED OBSERVATION AND DATA‐DRIVEN ANALYSIS OF INDIVIDUAL AND SOCIAL BEHAVIOR

The recent digital data revolution, including the advent of *internet‐of‐things* technologies and advancement of *machine learning* analytics (for definitions of technical terms, which are italicized, see Table 1), has opened the door for automated observations of human behavior at scales that have never before been possible (Lazer et al., 2009). One promising approach uses wearable devices, which allow precise spatiotemporal recording of activities of single individuals, dyadic interaction partners, or members of social groups (Mukhopadhyay, 2014). In the preschool context, lightweight and autonomous wearable devices can enable researchers to observe hundreds of children during free interactions in natural settings, without restrictions and with limited observational biases (e.g., Dai et al., 2020).

**TABLE 1:** Glossary of technical terms.

Accelerometer	A device that measures the force caused by an object's or person's acceleration/change in motion
Artificial neural networks (ANN)	A machine learning paradigm designed to mimic the processing power of a human brain
Bluetooth	A short‐range wireless technology that uses radio waves on a particular frequency to exchange data (range: up to 10 m/33 ft)
Ground truth	Data sample that has been collected by human observers used for training, testing, and validation of supervised mawchine learning algorithms
Gyroscope	A device that measures an object's or person's orientation and how fast this orientation changes over time
Internet of things	A system that appears as a local network of several communicating sensors and smart devices
Long short‐term memory	An ANN with several layers and feedback loops used in sequence learning settings
Machine learning	A subfield of artificial intelligence in computer science that aims to design algorithms that build on data to imitate human decision‐making with gradually improving accuracy
Radio‐frequency identification (RFID)	A wireless system that uses radio waves on various frequency bands; it consists of readers and tags, allowing to automatically identify and track tags attached to objects or people (range: passive systems up to 10 m/33 ft; active systems up to 100 m/328 ft)
Random forests	A machine learning method that builds up from an ensemble of random trees to solve classification or regression problems
Reality mining	A subfield of human dynamics that employs wearable devices to simultaneously follow multiple aspects of social and individual behavior in real‐world settings
Ultra‐wideband (UWB)	A wireless technology that uses low‐energy radio waves over a large portion of the radio spectrum to exchange data (range: up to 25 m/82 ft)

*Note*: These terms are related to the technological and analytical advances relevant to the automated observation and data‐driven analysis of individual and social behavior.

Since the first *reality mining* project used mobile phones as personal sensors to log traces of human behavior (Eagle & Pentland, 2006), a new research field capitalizing on this idea has emerged. Several projects have tracked individuals' movement with *accelerometers* and *gyroscopes* (Bayat et al., 2014; Karantonis et al., 2006), allowing fine‐grained measurement of acceleration, rotation, and body position. Other efforts have studied individuals as part of a social group, estimating their social interactions from timed proximity data using *Bluetooth* technology (Liu et al., 2013; Scherrer et al., 2008; Sekara & Lehmann, 2014), *ultra‐wideband* ranging (UWB), or *radio‐frequency identification* (RFID). These devices use radio signals to communicate and transmit data packages within a certain range. They record the identification of nearby devices, and either signal strength or time‐of‐flight can be used to approximate distance between devices. They have proven especially useful in social settings because they are relatively inexpensive and easily customizable to measure proximity, approach velocity, and patterns of initiating and terminating proximity (Banarjee et al., 2023; Cattuto et al., 2010; Scherrer et al., 2008). While early designs relied on centralized data collection settings that required a fixed laboratory setup, recently developed technologies (e.g., OpenBeacon, Obimon Kft.) are fully autonomous. This allows researchers to record participants' movement in any context, thereby taking observations out of the laboratory and into natural settings (Kiti et al., 2016; Ozella et al., 2021).

Although these novel technologies have opened a new chapter in social sciences, they come with challenges. Since automated data collection does not require human involvement, researchers need to verify the accuracy of the collected data. They can do so by establishing the so‐called *ground truth* during the early stages of data collection, either by synchronized video recordings (Elmer et al., 2019) or by involving experts on‐site to simultaneously collect data by hand or with tailored mobile applications (Altman et al., 2020; Dai et al., 2020). Comparing the automated data to the ground truth provides a quantitative measure of the accuracy of the collected data. Moreover, automated data collection is particularly powerful for capturing so‐called “dynamic microprocesses” of social interactions (Torrens & Griffin, 2013, p. 584), such as acceleration, approach velocity, and proximity, because it allows precise and scalable observations. However, extracting meaningful information from such simple measures requires thoughtful considerations. To precisely reconstruct social interactions from the recorded signals, machine learning methods have proved useful.

Machine learning techniques have been used to automatically detect and classify behavioral patterns and physical activities from accelerometer data (e.g., *random forests*, Ahmadi et al., 2020; *artificial neural networks*, Hagenbuchner et al., 2015). Moreover, sequence learning methods (e.g., *long short‐term memory*) have been applied to classify mutual proximity readings from pairs of RFID sensors into records of social interactions with precise time stamp and duration (Dai et al., 2020). While these methods cannot detect causal patterns in social behavior, their sensitivity to correlations allows them to solve certain inference tasks, sometimes even outperforming human assessments (Fujiwara et al., 2021; Kühl et al., 2022). For example, in one study, automated analysis of children's facial expressions was more accurate in detecting deception than were human observers (Bruer et al., 2020). Therefore, researchers have started using automated recordings and machine‐learning approaches to observe children's behavior in natural settings.

In the last decade, various automated data collection efforts have focused on children, seeking new approaches to track their early development of individual and collective behavior. On the individual level, in several studies, using accelerometers allowed researchers to quantify preschool childrn's level of physical activity and classify between broad types of activities in natural settings (e.g., walking vs. sitting, light vs. vigorous activity; Ahmadi et al., 2020; Hagenbuchner et al., 2015; Nilsen et al., 2019). In another study, movement and location data derived from a commercial UWB system (Ubisense, Inc.) were used to assess children's atypical movement patterns in an inclusive preschool classroom (Irvin et al., 2018). On the group level, RFID devices have been used in various preschool and school settings to observe the dynamic social networks of children through records of face‐to‐face interactions (e.g., preschool: Dai et al., 2022; Messinger et al., 2019; primary school: Cattuto et al., 2010; Stehlé et al., 2011).

These studies replicated well‐established findings about social dynamics in preschool groups (e.g., selective grouping by gender; Messinger et al., 2019) and shed new light on group formation strategies among preschoolers (Iacopini et al., 2023). Fine‐grained analyses of children's interactions in their typical environments showed that some classroom environments elicit more social interactions than others (e.g., building blocks vs. science area; Irvin et al., 2021) and that preschoolers' social competence is predicted by the duration of their interactions with their peers (Veiga et al., 2017). In some cases, sensors were also equipped with directional microphones to capture speech or record conversations among preschool peers (Dai et al., 2022; Fasano et al., 2021; Irvin et al., 2021).

These studies offer a unique opportunity to understand how preschoolers' social and linguistic capabilities co‐evolve (Dai et al., 2020). For example, in a study of inclusive classrooms, preschoolers with autism spectrum disorder engaged in fewer vocal interactions with others than their typically developing peers, but children's engagement with others may have depended on the specific social networks within the different groups (Fasano et al., 2021). These pioneering studies demonstrated the relevance of using wearable sensor technology and machine learning analytics for automated recording of behavior in children by identifying correlations that are difficult to capture with conventional data collection methods. However, researchers are just beginning to use these technologies to their full capacity for completely integrating the dimensions of space and time; the levels of individual, dyad, and group; and the different types of sensors to reach a more fine‐grained, large‐scale observation of children's behavior.

## THE GREAT BEYOND: INTEGRATION

As we have discussed, wearable sensors and machine learning analytics allow automated and precise measurements of an individual's position and velocity and a dyad's proximity and synchrony, as well as association patterns and overall coordination within a group. As a next step, researchers can use the technological and analytical advantages of this approach to integrate young children's movements with others in space and time into a coherent framework of social interaction (see Figure 1).

In our natural interactions with others, the dimensions of space and time are deeply intertwined. For example, affiliation makes us seek the proximity of another individual as well as coordinate our behavior with them, not just one or the other. Evidence suggests that homophily among preschool dyads can be detected both in velocity of approach and duration of proximity (Banarjee et al., 2023). Evidence also points to the importance of incorporating information on temporal changes in social networks to fully understand how individuals navigate groups and how groups form and disaggregate (Iacopini et al., 2023). Such detailed analyses are only possible with enough data that combine information about children's interactions with others in space and time.

Moreover, while observing single individuals in a particular context has been useful in understanding individual behavior, this approach limits our understanding of social phenomena, such as leader‐follower dynamics within children's groups. Concepts borrowed from complex systems theory offer a viable approach to such phenomena by interpreting social contacts as complex interaction networks, revealing underlying mechanisms explaining observed collective behavior (Daniel et al., 2019; Farmer & Farmer, 1996). A preschool peer group consists of individuals with specific skills and temperaments, of dyads with specific relationships, and of subgroups within the larger peer group. Observing any of these elements in isolation rather than as they interrelate limits our abilities to understand them more fully. Through large‐scale automated data collection, combined with machine‐driven behavior inference and reconstruction techniques, we can gain a more comprehensive understanding of the intertwined behaviors of individuals, dyads, and larger social groups within complex social networks.

Finally, to observe multiple aspects of social behavior simultaneously, some pioneering studies have already integrated different types of sensors, such as proximity sensors and audio recorders with automated language recognition, to get a more detailed picture of preschoolers' social interactions in natural settings (e.g., Altman et al., 2020). Integrating accelerometers, proximity sensors, and voice recorders into a single wearable device represents an initial step toward dynamically observing various aspects of human behavior in the same context with minimal intervention. Such detailed recordings of preschoolers' behavior in relation to their peers, aided by advanced machine learning methods, would allow researchers to classify more accurately the form and quality of social interactions.

## CONCLUSION

In this article, we have reviewed studies examining preschool children's affiliation and social dynamics in peer groups and, addressing the challenges of this research, have proposed an integrative framework based on wearable sensor technology and machine learning analytics. The fine‐grained and large‐scale data collection this approach allows would let researchers use children's movement with and in relation to their peers as a proxy for complex social group dynamics. The approach can also enhance our theoretical understanding of children's social development.

Adopting these novel technologies naturally leads to new challenges, including ethical concerns regarding data collection (Segura Anaya et al., 2018) and the privacy of underage participants (Datta et al., 2018). To address these issues, the field must standardize efforts to inform participants; obtain consent from legal guardians; and set up certified and safe computational infrastructures to collect, curate, clean, analyze, and store data (cf. Dai et al., 2022). More generally, developmental psychological research has recently started addressing issues related to overly selective and nonrepresentative samples (e.g., Henrich et al., 2010). For example, most studies we included in this review have been conducted with participants from Western societies (see Table S1). For many studies, there was only limited or no sociodemographic information available and if there was, it showed that the participants were not overly representative (e.g., studies of children who were predominantly White and middle class; studies of adults who were predominantly undergraduate students; see Table S1). Concerns about sample selection are often perpetuated by the way researchers traditionally design and conduct experiments. Parents are typically invited to visit child laboratories, where individual children are tested in paradigms that are often considerably different from their everyday experiences. Yet, findings from these settings are used to make assumptions about children's behavior and functioning outside this specific environment. These issues hinder the democratization of developmental science, preventing it from becoming diverse and feasible across cultures and contexts, as well as accessible, generalizable, and replicable. Our proposed approach to studying peer interactions may offer a more inclusive experimental strategy than traditional paradigms since it takes observations out of the laboratory and into natural settings. By relying on automated recordings via relatively inexpensive and unobtrusive wearable sensors, such observations may reduce the burden on resources and lessen implicit biases, addressing privacy concerns, and allowing broader applicability in both Western and non‐Western societies (see Kiti et al., 2016; Ozella et al., 2021). Researchers need to evaluate the feasibility of this framework and its applicability across a wider range of psychological constructs (e.g., infant‐caregiver attachment).

We believe our proposed framework will provide a more complex picture of young children's social interactions and relationships. Because our approach is unobtrusive and ecologically valid, highly automated, and thus more precise and less effortful than traditional data collection methods, it represents a scalable human technology. This framework provides a novel approach to reliably and accurately capturing social dynamics not only in preschool, but more generally in natural groups of various compositions (e.g., families) and across different ages. Our framework opens possibilities for scientific study to extend beyond the frame of basic research toward personalized skill assessment, training, and intervention among preschool children.

## FUNDING INFORMATION

Writing of this article was supported by the Austrian Science Foundation (FWF; project V‐893 to Lisa Horn) and a Postdoc Award of the Faculty of Psychology, University of Vienna (PA‐20/1/03) to Gabriela Markova. Márton Karsai acknowledges support from the DylNet ANR project funded by the Agence Nationale de la Recherche (ANR‐16‐CE28‐0013), the Horizon 2020/CHIST‐ERA project SAI, and the FWF (project I 5205‐N).

## Supporting information


Table S1.
