# Supplementary material for: An automated, data‐driven approach to children's social dynamics in space and time
Source: Child Dev Perspect. 2023 Dec 8;18(1):36–43. doi: 10.1111/cdep.12495 (PMC10953409; doi:10.1111/cdep.12495)
Supplement: Supplementary file 1 — Table S1. [file CDEP-18-36-s001.docx]

**Table for Supplemental Materials**

*Sociodemographic Information about the Empirical Studies*

| Afshordi, N. (2019). | Experiment 1:  Children; *N* = 48; 50% female; 4 years; *M*_age_ = 4.5 years; US urban sample (Boston); mainly middle class and white (79% European American, 15% Asian American, 6% African American)  Adults; *N* = 120; 46% female; *M*_age_ = 33.6 years; US sample, recruited via Amazon mTurk; no sociodemographic information  Experiment 2:  Children; *N* = 27; 48% female; 4 years; *M*_age_ = 4.55 years; no sociodemographic information |
| --- | --- |
| Ahmadi, M. N., Pavey, T. G., & Trost, S. G. (2020). | Children; *N* = 31; 39% female, 71% male; 3–5 years; Australian sample (Brisbane); no sociodemographic information |
| Altman, R. L., Laursen, B., Messinger, D. S., & Perry, L. K. (2020). | Children; *N* = 9; 56% female, 44% male; 2–3 years (at the outset of the study); *M*_age_= 30.33 months, *SD* = 2.45; US sample (Miami); 22% African Americans, 33% Anglo Americans, 33% Hispanic Americans, 11% with a mixed ethnic background; 78% using hearing aids or cochlear implants, 22% typically hearing |
| Banarjee, C., Tao, Y., Fasano, R., Song, C., Vitale, L., Wang, J., Shyu, M.-L., Perry, L. K., & Messinger, D. S. (2023). | Children; *N* = 77; 43% female; *M*_age_ = 48.26 months, *SD* = 7.47; US sample (Miami); 86% Hispanic, 14% non-Hispanic; 96% White, 3% Black, 1% multiracial; 61% with developmental disabilities, 39% typically developing |
| Bayat, A., Pomplun, M., & Tran, D. A. (2014). | Adults; *N* = 4; 50% female, 50% male; 29–33 years; US sample; no sociodemographic information |
| Bernieri, F. J., Reznick, J. S., & Rosenthal, R. (1988). | Children; *N* = 8; 50% female, 50% male; 14–18 months; tested with their mothers; US sample; no sociodemographic information |
| Bruer, K. C., Zanette, S., Ding, X. P., Lyon, T. D., & Lee, K. (2020). | Children 4–9 years; *N* = 158; US sample, low-income households;  maltreated group: *N* = 93; 49% female; *M*_age_ = 6.66, *SD* = 1.61; 69% Latino/a, 16% African American, 9% other, 6% biracial;  nonmaltreated group: *N* = 65; 54% females; *M*_age_ = 6.57, *SD* = 1.47; 82% Latino/a, 7% African American, 10% biracial |
| Cattuto, C., Van den Broeck, W., Barrat, A., Colizza, V., Pinton, J. F., & Vespignani, A. (2010). | Adults; *N* = 1,005; lab members and conference participants; EU sample (Turin, Berlin, Nice); no gender, age or sociodemographic information (data completely anonymized) |
| Chen, J., Lin, T.-J., Justice, L., & Sawyer, B. (2019). | Children; *N* = 485; 42% female; 34–70 months, *M*_age_ = 51.88, *SD* = 6.24; US sample; 78% Caucasian, 11% African American, 1% Asian, 9% other; from 64 early childhood special education (ECSE) inclusive classrooms; majority of mothers had at least some post-secondary education, the average level of annual household income was $40,001–$60,000 |
| Cirelli, L. K., Wan, S. J., & Trainor, L. J. (2016). | Children; *N* = 48; 50% female; 14 months; *M*_age_ = 14.7 months; *SD* = 0.3 months; Canadian sample (Hamilton/Ontario and surrounding neighborhoods); of mixed ethnicities; from homes where English was spoken over 50% of the time |
| Dai, S., Bouchet, H., Karsai, M., Chevrot, J. P., Fleury, E., & Nardy, A. (2022). | Children; *N* = 174; from three classes (small class: 3-4 years; middle  class: 4–5 years; grand class: 5–6 years); French sample; children’s age, gender, socio-demographic characters, socialization, verbal, linguistic skills, family cultural, education and employment characteristics are shared upon request  Adults; *N* = 32; French sample; no gender, age or sociodemographic information |
| Dai, S., Bouchet, H., Nardy, A., Fleury, E., Chevrot, J. P., & Karsai, M. (2020). | Children; *N* = 165; from two classes (middle class: 4–5 years; grand class: 5–6 years); French sample; no gender or sociodemographic information  Adults; *N* = 25; no gender, age or sociodemographic information |
| Daniel, J. R., Santos, A. J., Fernandes, C., & Vaughn, B. E. (2019). | Children; *N* = 169; 47% female; from 6 classrooms divided by age: 3 years (< 48 months at the start of the academic year), 4 years (48–60 months), 5 years (60–72 months); Portuguese sample (Lisbon area); middle class; no sociodemographic information |
| Eagle, N., & Pentland, A. (2006). | Adults; *N* = 100; US sample (Cambridge/Massachusetts); no gender, age or sociodemographic information |
| Elmer, T., Chaitanya, K., Purwar, P., & Stadtfeld, C. (2019). | Experiment 1:  Adults; *N* = 11; Swiss sample; university staff and students; no gender, age or sociodemographic information  Experiment 2:  Adults; *N* = 73; 37% female; Swiss sample; university students; no age or sociodemographic information |
| Farmer, T. W., & Farmer, E. M. (1996). *Exceptional Children*, *62*, 431-450. | Children; *N* = 79; 50.6% female; US sample; 74.7% White, 22.8% African American, 2.5% other races; 49.4% receiving general education, 21.5% classified as academically gifted, 8.9% having learning disabilities, 5% having emotional and behavioral disorders, 15.2% with unknown educational classification; no age information |
| Fasano, R. M., Perry, L. K., Zhang, Y., Vitale, L., Wang, J., Song, C., & Messinger,  D. S. (2021). | Children; *N* = 56; 39.3% female; *M*_age_ = 50.14 months, *SD* = 7.06; US sample; 87.5% Hispanic White, 10.7% non-Hispanic White, 1.8% Hispanic Black; 28.6% with autism spectrum disorder, 33.9% with developmental delay, 37.5% typically developing; children in inclusion classrooms; no sociodemographic information |
| Fawcett, C., & Tunçgenç, B. (2017). | Children; Swedish sample (Uppsala); volunteers from a medium-sized university town; no sociodemographic information  12-months-olds: *N* = 33, 51.5% female; *M*_age_ = 12.04 months, SD = 0.23  5-month-olds: *N* = 32, 46.9% female; *M*_age_ = 14.94 months, SD = 0.24 |
| Feldman, R. (2012). | Children; tested at birth, 3, 6, 12, and 24 months, 5 and 10 years; tested with their mothers and fathers; no sample size, gender or sociodemographic information |
| Fujiwara, K., Bernhold, Q. S., Dunbar, N. E., Otmar, C. D., & Hansia, M. (2021). | Adults; *N* = 111; 68% female; *M*_age_ = 19.87, *SD* = 1.50; US sample (West coast); no sociodemographic information |
| Fujiwara, K., Kimura, M., & Daibo, I. (2020). | Study 1:  Adults; *N* = 84; 45.25% female; *M*_age_ = 18.77, SD = 1.07; Japanese sample; undergraduate university students; no sociodemographic information  Study 2:  Adults; *N* = 150; 66.7% female; *M*_age_ = 19.09, SD = 1.05; Japanese sample; undergraduate university students; no sociodemographic information |
| Hagenbuchner, M., Cliff, D. P., Trost, S. G., Van Tuc, N., & Peoples, G. E. (2015). | Children; *N* = 11; 3–6 years, *M*_age_ = 4.8, *SD* = 0.87; mean BMI = 15.9 kg/m^2^, *SD* = 1.0; 9.1% overweight; Australian sample (Wollongong); no gender or sociodemographic information |
| Hartup, W. W., Laursen, B., Stewart, M. I., & Eastenson, A. (1988). | Children; *N* = 53; 50.9% female; 3.33–5.33 years; *Mdn* = 4.25 years; US sample; 79.2% White, 3.8% Black, 17% other minority groups; middle and mixed class |
| Hoch, J. E., Ossmy, O., Cole, W. G., Hasan, S., & Adolph, K. E. (2021). | Children; *N* = 30; 53.3% female; 12.89–19.53 months, *M*_age_ = 15.86; US urban sample (New York City); 60% White, 3.3% Black, 6.7% Asian, 23.3% multiple races, 6.7% race not reported; 20% Hispanic or Latino, 3.3% ethnicity not reported; tested with their mothers: 28.29–48.68 years, *M*_age_ = 34.85; family income $44,634–$214,946, *M* = $116,191 |
| Howes, C. (1990). | Children; *N* = 45; 53.3% female; tested in laboratory kindergarten and third grade primary school; US sample; 66.7% Euro-American, 13.3% Afro-American, 11.1% Hispanic, 8.9% Asian-American; 72% living in two-parent-families; no age information; mother’s educational level *Mdn* = 15 years, range = eighth grade to Ph.D. |
| Iacopini, I., Karsai, M., Barrat, A. (2023). | Dataset 1:  Adults, *N* = 706; Danish sample (Copenhagen); freshman university students; no gender, age or sociodemographic information (data completely anonymized)  Dataset 2:  Children; *N* = 174; from three classes (small class: 3-4 years; middle  class: 4–5 years; grand class: 5–6 years); French sample; children’s age, gender, socio-demographic characters, socialization, verbal, linguistic skills, family cultural, education and employment characteristics are shared upon request  Adults; *N* = 32; French sample; no gender, age or sociodemographic information |
| Irvin, D. W., Crutchfield, S. A., Greenwood, C. R., Kearns, W. D., & Buzhardt, J. (2018). | Children; *N* = 3; 33.3% female; 3.08–5.25 years; US sample (Midwest); 66.7% typically developing, 33.3% with medical diagnosis of Down syndrome; no sociodemographic information |
| Irvin, D. W., Luo, Y., Huffman, J. M., Grasley-Boy, N., Rous, B., & Hansen, J. H. L. (2021). | Children; *N* = 3; 100% male; 2.92–3.08 years; US urban sample (Southern state); 66.7% White, 33.3% bi-racial; 66.7% typically developing, 33.3% at-risk for a disability; inclusive classroom; mothers of all children college educated |
| Karantonis, D. M., Narayanan, M. R., Mathie, M., Lovell, N. H., & Celler, B. G. (2006). | Adults; *N* = 6; 22-60 years; US sample; no gender or sociodemographic information |
| Kiti, M. C., Tizzoni, M., Kinyanjui, T. M., Koech, D. C., Munywoki, P. K., Meriac,  M., Cappa, L., Panisson, A., Barrat, A., Cattuto, C., & Nokes, D. J. (2016). | Children & adults; *N* = 5 households (a household encompasses several related families living in distinct houses within the same compound and reporting to one head); between <1 and ≥50 years; Kenyan sample; no gender or sociodemographic information |
| Kühl, N., Goutier, M., Baier, L., Wolff, C., & Martin, D. (2022). | Adults; *N* = 44; 45.5% female, 54.5% male; *M*_age_ = 26 years, *SD* = 9.6; German sample; 91% university students; no sociodemographic information |
| Latif, N., Barbosa, A. V., Vatiokiotis-Bateson, E., Castelhano, M. S., & Munhall, K. G. (2014). | Adults; *N* = 62; 58.1% female; *M*_age_ = 21.2 years; Canadian sample; undergraduate university students; no sociodemographic information |
| Liberman, Z., & Shaw, A. (2019). | Study 1-3:  Children; *N* = 742; 3–11 years; US sample (Chicago); tested at a science museum; no gender or sociodemographic information  Study 4:  Children; *N* = 158; 3–11 years; US sample; tested at a science museum and at a zoo in Santa Barbara; no gender or sociodemographic information |
| Liu, S., Jiang, Y., & Striegel, A. (2013). | Adults; *N* = 196; US sample (Indiana); freshmen university students; no gender, age or sociodemographic information |
| Martin, C. L., Kornienko, O., Schaefer, D. R., Hanish, L. D., Fabes, R. A., & Goble, P. (2013). | Children; *N* = 292; 46.6% female; *M*_age_ = 4.3 years; US sample; 69% Mexican or Mexican American, 8% Anglo-American, 7% African American, 2% Asian, 1% Native American, 13% other or unknown ethnicities; 60% primarily spoke Spanish; 45% from two-parent married families; low socioeconomic status (82% earned below $30,000 per year) |
| Masters, J. C., & Furman, W. (1981). | Children; *N* = 94; 4–5 years; White; middle class; no gender information |
| Messinger, D. S., Prince, E. B., Zheng, M., Martin, K., Mitsven, S. G., Huang, S., Stölzel, T., Johnson, N., Rudolph, U., Perry, L. K., Laursen, B., & Song, C. (2019). | Children; *N* = 16; 50% female; *M*_age_ = 61.8 months, *SD* = 8.1; German sample (Southern Germany); no sociodemographic information |
| Nilsen, A. K. O., Anderssen, S. A., Ylvisaaker, E., Johannessen, K., & Aadland, E. (2019). | Children; *N* = 1308; 2.7–6.5 years; Norwegian sample (Sogn og Fjordane); no gender or sociodemographic information |
| Ozella, L., Paolotti, D., Lichand, G., Rodríguez, J. P., Haenni, S., Phuka, J., ... & Cattuto, C. (2021). | Children & adults; *N* = 147; between <10 and >18 years; Malawi rural sample (Mdoliro village, Dowa district); majority Chewas ethnic group; majority Christians |
| Piek, J. P., Dawson, L., Smith, L. M., & Gasson, N. (2008). | Children; *N* = 33; 48.5% female; 6–11.5 years; *M*_age_= 8.5 years, *SD* = 1.9; Australian sample; low risk, no diagnoses of neurological disorders; no sociodemographic information |
| Rabinowitch, T. C., & Knafo-Noam, A. (2015). | Children; *N* = 148; 62.2% female; 8 years, *M*_age_ = 8.6 years, *SD* = 3.6 months; Israeli sample; no sociodemographic information |
| Santos, A. J., Daniel, J. R., Fernandes, C., & Vaughn, B. E. (2015). | Children; *N* = 240; 50% female; 3–5 years; Portuguese sample (Lisbon); middle class; families had European backgrounds |
| Sekara, V., & Lehmann, S. (2014). | Adults; *N* = 134; Danish sample; university students from 4 study majors (>93% of all students per study major); no gender, age or sociodemographic information |
| Stehlé, J., Voirin, N., Barrat, A., Cattuto, C., Isella, L., Pinton, J. F., Quaggiotto, M., Van den Broeck, W., Régis, C., Lina, B., & Vanhems, P. (2011). | Children; *N* = 232; 6–12 years; French sample (Lyon); no gender or sociodemographic information  Adults; *N* = 10; French sample (Lyon); no gender, age or sociodemographic information |
| Torrens, P. M., & Griffin, W. A. (2013). | Children; *N* = 84; *M*_age_ = 44.9 months, *SD* = 0.90; US sample (Southwestern metropolitan area); 58% European American, the remaining being Asian-, Mexican-, and African American in descending order; 69% from two-parent households with an average household annual income of US $116,000; no gender information |
| Tremblay, A., Strain, P. S., Hendrickson, J. M., & Shores, R. E. (1981). | Children; *N* = 61; about 50% females; 3–5.75 years; US sample; about 50% Black, about 50% White, 1 biracial child, 1 oriental child; lower-middle to upper-middle class |
| Tunçgenç, B., & Cohen, E. (2016). | Children; *N* = 102; 52% female; 84.1–139.34 months, *M*_age_ = 105.25; mixed ethnicities; middle-class |
| Veiga, G., de Leng, W., Cachucho, R., Ketelaar, L., Kok, J. N., Knobbe, A., Neto, C., & Rieffe, C. (2017). | Children; *N* = 73; 39.7% female; 46-68 months, *M*_age_=56.48, *SD* = 4.26; Portuguese sample; 86% Portuguese, 5% European, 3% African, 2% Brazilian; primarily middle class background; predominant level of maternal education was secondary education |
